# Transforaminal Endoscopic Lumbar Lateral Recess Decompression for Octogenarian Patients

**DOI:** 10.3390/jcm13020515

**Published:** 2024-01-17

**Authors:** Yong Ahn, Jun-Hyeok Jung

**Affiliations:** Department of Neurosurgery, Gil Medical Center, Gachon University College of Medicine, Incheon 21565, Republic of Korea; 18114@gilhospital.com

**Keywords:** endoscopic, lateral recess, lumbar stenosis, octogenarians, radiculopathy, transforaminal

## Abstract

The incidence of radiculopathy due to lumbar spinal stenosis has been on the increase in the aging population. However, patients aged ≥ 80 years hesitate to undergo conventional open surgery under general anesthesia because of the risk of postoperative morbidity and adverse events. Therefore, less invasive surgical alternatives are required for the elderly or medically handicapped patients. Transforaminal endoscopic lumbar lateral recess decompression (TELLRD) may be helpful for those patients. This study aimed to demonstrate the efficacy of TELLRD for treating radiculopathy in octogenarian patients. A total of 21 consecutive octogenarian patients with lumbar foraminal stenosis underwent TELLRD between January 2017 and January 2021. The inclusion criterion was unilateral radiculopathy, which stemmed from lumbar lateral recess stenosis. The pain source was verified using imaging studies and selective nerve blocks. Full-scale lateral canal decompression was performed using a percutaneous transforaminal endoscopic approach under local anesthesia. We found the pain scores and functional status improved significantly during the 24-month follow-up period. The clinical improvement rate was 95.24% (20 of 21 patients) with no systemic complication. In conclusion, endoscopic lateral recess decompression via the transforaminal approach is practical for octogenarian patients.

## 1. Introduction

As people live longer and have more complex lifestyles in modern society, adequate treatment options for degenerative spinal disease have become primary medical issues among elderly patients. Despite extensive conservative treatments, lumbar lateral recess stenosis (LRS) often results in unbearable radicular leg pain and requires surgical treatment. LRS is defined as the narrowing of the sides of the tubular passageway between the superior articular process (SAP) and the posterior vertebral margin [1,2,3]. Narrowing of the lateral passage may be caused by facet arthropathy, usually combined with hypertrophic ligamentum flavum (LF), redundant disc, and shoulder osteophyte [4,5]. The chronic impingement of the traversing nerve root (TNR) at the lateral spinal canal may elicit radicular symptoms and signs. If extensive conservative therapies fail to relieve the radicular pain, a decisive surgical treatment should be considered. Open laminectomy with or without fusion has been regarded as the standard surgical technique for LRS [6,7]. Nevertheless, opting for such open surgery under general anesthesia may cause considerable perioperative morbidity for elderly patients with medical problems. Some studies have reported a higher risk of perioperative complications in geriatric patients [8,9,10,11,12,13]. Several studies have reported harmful effects of general anesthesia in the elderly, such as cardiovascular or pulmonary dysfunction and impaired cognitive function [14,15,16,17,18]. As for spinal disorders, surgery in patients aged > 80 years has higher medical risks and more extended periods of hospital stay than surgery in younger patients [19,20,21,22]. Despite these challenges, some authors have reported that spine surgery for octogenarian patients is worthwhile, with acceptable complication rates [23,24,25,26]. Nonetheless, a less invasive and safer alternative surgical technique is needed for octogenarian patients with lumbar degenerative diseases.

A transforaminal, full-endoscopic approach enables spine surgeons to perform any surgical procedures under local anesthesia. Transforaminal endoscopic lumbar lateral recess decompression (TELLRD) is a minimally invasive option to treat radiculopathy with LRS. Notably, some studies have reported the transforaminal endoscopic decompression technique for LRS using various surgical devices, including endoscopic punches, trephines, and burrs [27,28,29,30,31,32,33,34,35]. Under local anesthesia, TELRD involves full-endoscopic lateral spinal canal decompression through a percutaneous tissue-preserving transforaminal approach. Therefore, this technique may be a viable option for those at risk of developing complications associated with open surgery under general anesthesia.

Although there are existing technique case series and cohort studies on TELLRD or similar technologies, clinical cohort studies on TELLRD in super-aged (≥80 years) patients have been lacking. Therefore, this study aimed to evaluate the surgical outcomes of TELLRD in octogenarian patients with LRS and explore the technical considerations crucial for achieving successful results.

## 2. Materials and Methods

### 2.1. Patient Population

The longitudinal data were prospectively entered into the database, and the records were retrospectively reviewed. Data were collected from 21 consecutive patients aged ≥ 80 years who underwent TELLRD between January 2017 and January 2021. This study was approved by the institutional ethical committee (GDIRB 2022-210), and written informed consent was obtained from the patients. The inclusion criteria for this surgery and study were as follows: (1) patients aged ≥ 80 years with unilateral radicular leg pain or neurogenic claudication, regardless of the presence of back pain; (2) definitive LRS confirmed using both computed tomography (CT) and magnetic resonance imaging (MRI); (3) the pain source verified by previous nerve root block; and (4) failure of extensive conservative treatments for at least six weeks. LRS was defined as the anteroposterior diameter of the lateral recess < 4 mm with or without herniated disc in the imaging studies [22]. The exclusion criteria included cauda equina syndrome, grade 2 or higher spondylolisthesis, disc herniation without LRS, severe central stenosis, and coexisting pathological conditions, such as systemic neuropathy, infection, and spinal tumors.

### 2.2. Surgical Procedure

**Figure 1:**
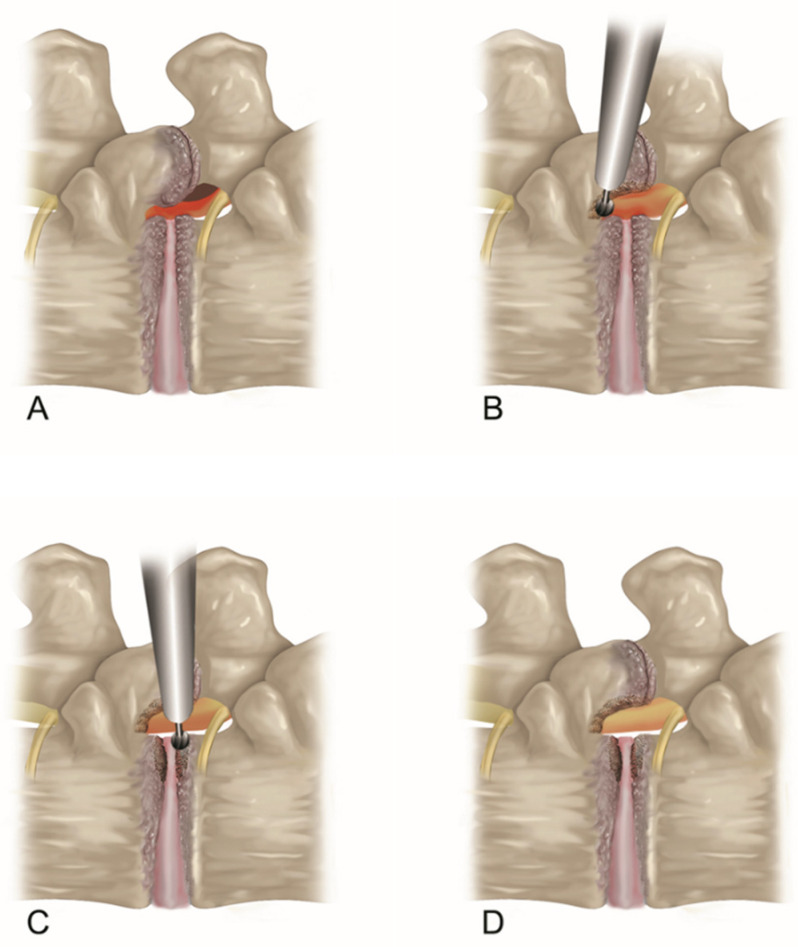
Schematic pictures of TELLRD. (**A**) Lumbar lateral recess stenosis. The TNR is compressed by the hypertrophied SAP, thickened LF, and pedicle. (**B**) Transforaminal dorsal decompression by resecting SAP, LF, and part of the pedicle using endoscopic burrs and punches at the lateral recess. (**C**) Transforaminal ventral decompression by removing shoulder osteophytes and redundant disc using endoscopic burrs and forceps to release the TNR. (**D**) Endpoint of the full-scale decompression of the lateral spinal canal. TELLRD, transforaminal endoscopic lumbar lateral recess decompression; TNR, traversing nerve root; SAP, superior articular process; LF, ligamentum flavum.

The surgical procedure was primarily based on a previously demonstrated TELLRD method [33]. This full-endoscopic decompression procedure was performed based on three steps: (1) the percutaneous transforaminal approach guided by fluoroscopy, (2) endoscopic bone resection using various burrs and punches, and (3) endoscopic soft tissue decompression using forceps and micropunches (Figure 1). For adequate conscious sedation, intramuscular midazolam (0.05 mg/kg) and intravenous fentanyl (0.8 μg/kg) were administered on call, and additional dosages could be administered according to the patient’s monitoring and surgeon’s needs. The patient was kept comfortably on a radiolucent spinal table in the prone position, with the hips and knees flexed.

#### 2.2.1. Transforaminal Approach

**Figure 2:**
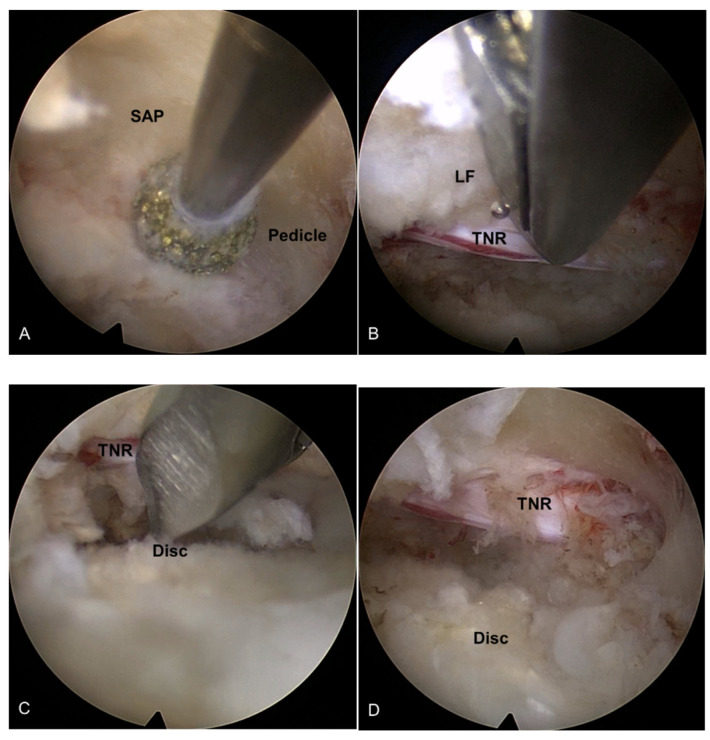
Intraoperative endoscopic pictures TELLRD. (**A**) Bony unroofing using endoscopic burrs and punches. The hypertrophic SAP and part of the pedicle were undercut using an endoscopic burr (L4–L5, left). (**B**) Ventral decompression with removal of thickened LF using endoscopic punches. (**C**) Dorsal decompression with removal of redundant disc and shoulder osteophytes using endoscopic burrs and punches. (**D**) Final endoscopic view showing the released TNR. TELLRD, transforaminal endoscopic lumbar lateral recess decompression; SAP, superior articular process; LF, ligamentum flavum; TNR, traversing nerve root.

The first step of TELLRD was transforaminal docking of the working sheath, exposing the foraminal structures and the surface of the facet joint. The skin entry point and the approach angle were determined based on preoperative imaging studies. Considering the properties of the endoscopy and related instruments, the primary approach angle can be recommended at approximately 45° or higher for full-scale decompression of the lateral side of the spinal canal. An 18-gauge spinal needle was advanced posterolaterally to the surface of the disc or the vertebral body close to the lateral side of the spinal canal and lateral recess. During this approach, fluoroscopic control ensured that the exiting nerve root (ENR) was not irritated by the instruments. The needle, firmly engaged in the foramen, was then substituted with a guidewire. A tapered obturator was inserted over the guidewire and advanced into the foramen with gentle pressure. Once the obturator was securely positioned in the foramen (not in the disc), a bevel-ended working sheath was run over the obturator with the sharp end directed opposite the ENR and placed on the undersurface of the facet joint. Following the withdrawal of the obturator, an ovoid working channel endoscope was inserted. Ideally, the bevel-ended working sheath should be securely engaged in the foramen, and the foraminal anatomy should be visualized, including the SAP, ENR, pedicle, and redundant disc (outside-in approach; Figure 2A).

#### 2.2.2. Endoscopic Bone Work

The early step of the lateral recess decompression focused on resecting the hypertrophic SAP with continuing pedicle and the medially-located inferior articular process (IAP) that compresses the traversing nerve root. The initial endoscopic view included the disc and the lateral surface of the SAP. Specific endoscopic burrs and micropunches were used for precise bone resection. Various types of burrs were applied for sufficient bone resection; round or side-cutting, straight or articulating, spanning between the upper and lower pedicle margins. The lateral and ventral portions of the hypertrophic SAP and IAP were systemically undercut from pedicle to pedicle and from lateral to the medial pedicular line until the ligamentum flavum (LF) was sufficiently exposed. During this process, a part of the pedicle was also resected until the epidural space with the traversing nerve root (TNR) was clearly defined. This point can be the typical landmark of the lateral aspect of the spinal canal. After confirming the TNR behind the pedicle, the exposed LF was performed. The lateral bone window was made large enough to facilitate subsequent soft tissue decompression. Bleedings from the bone surface and epidural space were controlled using a steerable radiofrequency (RF) coagulator tip and hemostatic agents (Figure 2B).

#### 2.2.3. Endoscopic Soft Tissue Decompression

After adequate lateral bony unroofing and widening, soft tissues, such as thickened LF, redundant disc, and shoulder osteophyte, were removed to alleviate compression on the TNR. Dorsolateral decompression was initiated by removing the hypertrophic LF with micropunches, small Kerrison punches, and semiflexible forceps with steerable RF coagulator tips. As the LF was gradually removed, the TNR and dural sac were also exposed. The decompression process required delicate tissue dissection under endoscopic visualization, and hydrostatic irrigation pressure was employed to aid dissection between the LF and neural tissues. The TNR was released from the axillary portion to the level of the inferior pedicle (Figure 2B). After sufficient dorsal decompression, a ventral decompression was performed. The shoulder osteophytes and redundant discs were removed using micropunches and burrs. The working sheath and endoscope were further advanced into the epidural space ventral to the dural sac. As the decompression progressed, the TNR became exposed and released (Figure 2C). This ventral work can be gradually performed from the lateral side to the midline, even to the contralateral side, as required. At this point, the surgeon can also encounter epidural or bone bleeding that interferes with the surgical field, which should be controlled using RF and hemostatic agents.

#### 2.2.4. The Target Point of the Decompression

**Figure 3:**
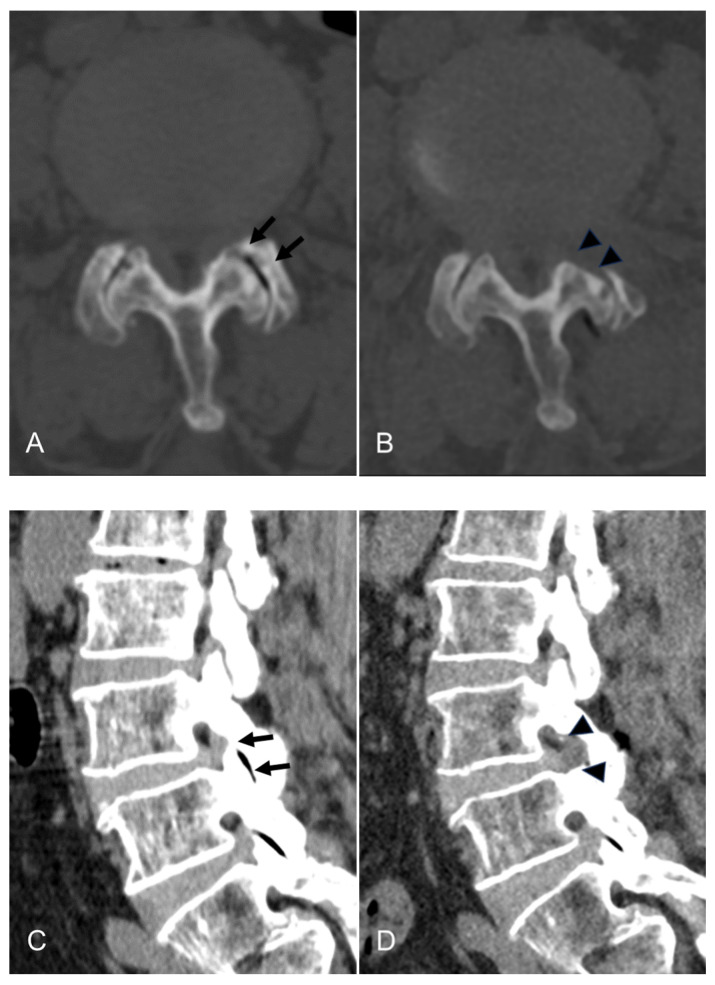

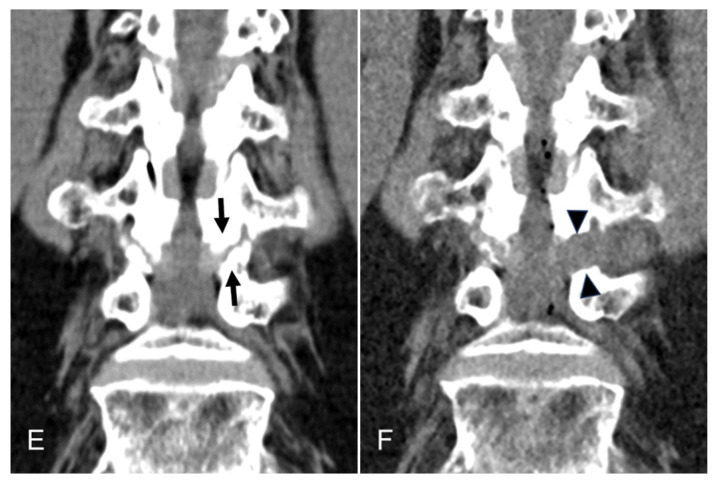
An illustrative case of an 81-year-old female patient treated with TELLRD. (**A**) Preoperative axial CT showing central and lateral recess stenosis at the L3–L4 level (arrows). (**B**) Postoperative axial CT showing lateral spinal canal decompression following undercutting of the hypertrophic SAP and LF compressing the TNR (arrowheads). (**C**) Preoperative sagittal CT showing lateral recess stenosis at the L3–L4 level (arrows). (**D**) Postoperative sagittal CT showing lateral spinal canal decompression following undercutting of the hypertrophic SAP and LF compressing the TNR (arrowheads). (**E**) Postoperative coronal CT showing lateral recess stenosis at the L3–L4 level (arrows). (**F**) Postoperative coronal CT showing lateral spinal canal decompression following undercutting the SAP and LF compressing the TNR (arrowheads). TELLRD, transforaminal endoscopic lumbar lateral recess decompression; SAP, superior articular process; LF, ligamentum flavum; TNR, traversing nerve root.

The endpoint of TELLRD was determined by identifying the released TNR with the dural sac from the axillary to the inferior pedicle level. Successful neural decompression was confirmed by observing the strong pulsation of the nerve in synchronization with the patient’s heartbeat and soft mobilization upon probing (Figure 2D). After adequate hemostasis, a sterile dressing was applied with a one-point subcutaneous suture. The patient was monitored for at least three hours to detect any adverse events and then discharged within 24 h. When required, postoperative imaging studies were conducted for precise pathological assessment (Figure 3).

### 2.3. Outcome Evaluation and Statistical Analysis

Data from a two-year follow-up were collected during periodic outpatient office visits and telephone surveys. Clinical outcomes were assessed using the visual analog scale (VAS) and Oswestry disability index (ODI) [36]. The global effects were evaluated using the modified MacNab criteria [37]: excellent (free of pain, no restriction of activity), good (occasional non-radicular discomfort, presenting symptom relief), fair (improved functional capacity, but still handicapped), or poor (insufficient improvement, further operative intervention required). Perioperative data, including operative time, length of hospital stay, and complications, were documented.

Statistical analyses were performed by an independent statistician using SPSS (version 14.0; SPSS Inc., Chicago, IL, USA). Pre and postoperative clinical data were compared using repeated-measures analysis of variance and paired *t*-tests. *p* < 0.05 was considered to be statistically significant.

## 3. Results

This study enrolled 21 patients (12 women, 9 men) with a mean age of 82.86 years (range, 81–88). The medical comorbidities were documented in 21 patients and included hypertension (*n* = 11, 52.38%), diabetes (*n* = 9, 42.86%), coronary heart disease (*n* = 7, 33.33%), Parkinson’s disease (*n* = 3, 14.29%), and cognitive problems (*n* = 2, 9.52%). The operated levels were L3–L4 in 6 (28.57%), L4–L5 in 10 (47.62%), and L5–S1 in 5 (23.81%) patients (Table 1). The mean operative duration was 59.67 min (range, 33–85 min). The mean postoperative hospital stay was 1.8 days (range, 1–6 days).

**Figure 4:**
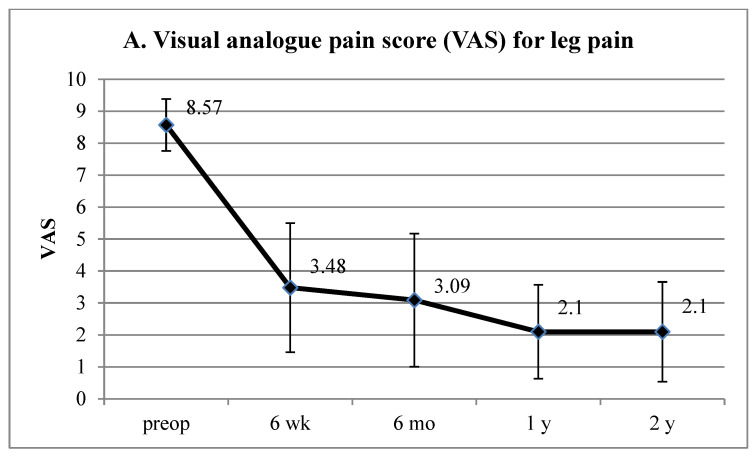

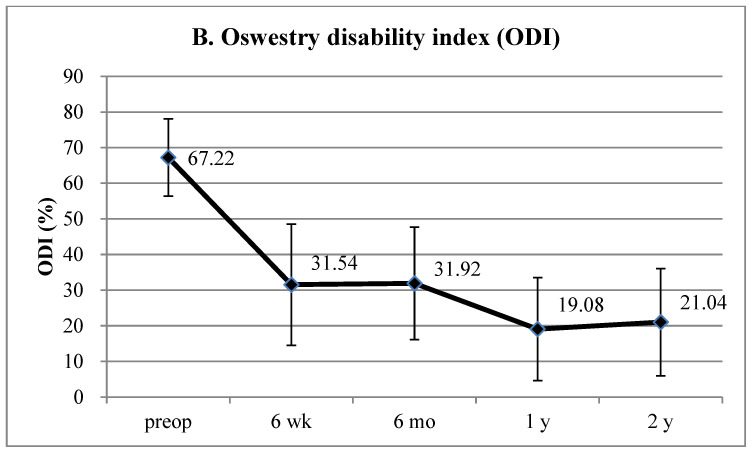
Clinical outcomes of TELLRD for octogenarian patients. (**A**) VAS pain score for radicular pain preoperatively and at 6 weeks, 6 months, 1 year, and 2 years postoperatively. (**B**) ODI scores preoperatively and at 6 weeks, 6 months, 1 year, and 2 years postoperatively. TELLRD, transforaminal endoscopic lumbar lateral recess decompression; VAS, visual analog scale; ODI, Oswestry disability index.

The VAS score (mean ± SD) for the lumbar radiculopathy significantly improved from 8.57 ± 0.81 preoperatively to 3.48 ± 2.02, 3.09 ± 2.08, 2.10 ± 1.47, and 2.10 ± 1.56 at six weeks, six months, one year, and two years postoperatively, respectively (*p* < 0.001, Figure 4A). Additionally, the ODI score (mean ± SD) improved from 67.22 ± 10.85% preoperatively to 31.54 ± 17.03%, 31.92 ± 15.80%, 19.08 ± 14.46%, and 21.04 ± 15.04% at six weeks, six months, one year, and two years postoperatively, respectively (*p* < 0.001; Figure 4B).

**Figure 5:**
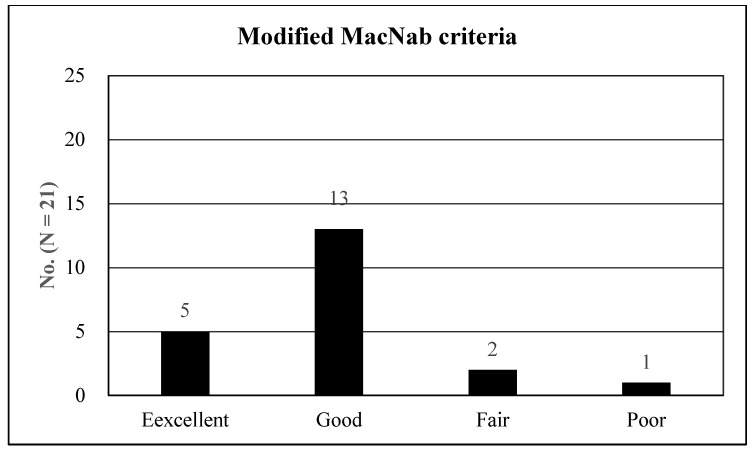
The four-point overall outcomes based on the modified MacNab criteria: the procedure outcomes were excellent in 5 patients (23. 81%), good in 13 (61.90%), fair in 2 (9.52%), and poor in 1 (4.76%). Therefore, the success rate was 85.71%, and the clinical improvement rate was 95.24%.

The overall outcomes according to the 4-point outcome scale (modified MacNab criteria) were excellent, good, fair, and poor in 5 (23.81%), 13 (61.90%), 2 (9.52%), and 1 (4.76%) patient, respectively. Therefore, the rate of symptomatic improvement was 95.24% (Figure 5). Of the 21 patients, one with poor outcomes experienced sustained radicular pain and postoperative flares. The patients were managed with open lumbar laminectomy. One minor dural tear was noted but repaired intraoperatively using an adhesive, closing sheet and fibrin sealant. No other significant postoperative complications were observed, such as hematoma or infection. Furthermore, no segmental instability was reported in the follow-up radiological studies.

Operative data and the global outcomes were compared with patients younger than 80 years old who underwent TELLRD during the same period. The success rate based on the modified Macnab criteria and complication rate were similar between the two age groups (Table 1).

## 4. Discussion

### 4.1. Interpretation of Clinical Results

Our findings indicate clinical efficiency in terms of pain intensity, functional indices, and overall results. The mean VAS score for the radiculopathy improved by 6.48 at the final evaluation (*p* < 0.001), and the mean ODI reduced by 46.17 at the last follow-up (*p* < 0.001). Clinical relevance is often defined as more than a 50% reduction in the VAS score [38] and more than a 30% improvement in the ODI [39,40]. Therefore, TELLRD for octogenarian patients in our series resulted in relevant clinical outcomes. Compared with the published data on the outcomes of TELLRD for all age groups [33], in which the mean VAS reduction was 6.36 and the mean ODI reduction was 46.5, our data for this series also revealed comparable results. The modified McNab criteria, complication, and revision surgery were also comparable to those of younger patients who underwent TELLRD during the same period (Table 1). Given that most elderly patients have concurrent medical problems and risks associated with general anesthesia, the clinical relevance of endoscopic procedures under local anesthesia can prevent the systemic adverse events of open surgery under general anesthesia.

### 4.2. Pros and Cons of TELLRD (Why Is Endoscopic Foraminotomy Feasible under Local Anesthesia?)

There are several reasons that TELLRD under local anesthesia may be effective for elderly or medically compromised patients at risk for general anesthesia. First, the transforaminal approach is adequate for decompression of the lateral zone of the spinal canal while avoiding the vulnerable central zone. The dural membrane is exposed minimally, and the hydrostatic pressure or mechanical irritation to the dural sac may be minimal during the decompression process. Patients can tolerate the surgical procedure with minimal dural irritation signs in an aware status. Second, not only the usual dorsal decompression, but an additional ventral canal decompression is feasible through the transforaminal approach. Ventrally protruding osteophytes and discs can be decompressed without burdensome dural sac retraction. Therefore, the lateral canal decompression effect may be enhanced compared to the conventional posterior approach. Third, a percutaneous approach with a minimal stab incision may reduce musculoskeletal tissue damage and the risk of complications, including muscle atrophy, segmental instability, surgical site infection, or hematoma. Fourth, the short operative time under local anesthesia may facilitate postoperative recovery without the risk of general anesthesia in geriatric patients. Finally, endoscopic surgical techniques have remarkably evolved owing to the development of specialized surgical devices, such as various endoscopic burrs, steerable or articulating forceps, and micropunches.

However, the steep learning curve and technical limitations may be the entry barriers to the completion of this local endoscopic technique [41]. A relevant and reproducible outcome can be obtained only after achieving technical proficiency. The standard spine surgeons have limited opportunities to learn and practice endoscopic spine procedures during their residence or training period. A systemic learning process and extensive clinical experience are mandatory to apply endoscopic spine procedures in actual practice. Therefore, the clinical applications of TELLRD should be carefully considered.

Although the procedure is relatively safe, TELLRD performed under local anesthesia may cause adverse events. First, approach-related or manipulation-related pain may disturb the process or even cause surgical failure. Therefore, this procedure requires excellent technical proficiency to prevent procedural pain. Second, the adverse effects of sedatives and local anesthetics should not be ignored. Elderly patients in the prone position may be vulnerable to drugs for conscious sedation. Fluctuations in blood pressure, heart rate, and respiration may affect surgical outcomes. Thorough monitoring of the patient’s vital signs and delicate management of the sedation status are essential to keep the patient stable during the procedure. Finally, specific complications related to the transforaminal approach, such as dural tears, incomplete decompression, and postoperative dysesthesia should be considered. A long learning curve is required to achieve technical proficiencies. Complete mastering of the surgical technique and anatomy is essential for clinical success.

### 4.3. Technical Pearls to Success

For elderly or medically handicapped patients, the procedure should be conducted smoothly in a limited time under proper anesthesia while keeping a safe range of vital signs. Therefore, practical and valuable keys to success are required. First, the typical landing point is the midpoint between the disc space and the inferior pedicle, corresponding to the lateral recess. Second, endoscopic bony unroofing and soft tissue decompression should cover the lateral aspect of the spinal canal, from the proximal margin of the LF to the distal margin of the LF and mid-pedicular level. Undercutting of the facet joint should be enough to expose the lateral aspect of the LF fully. Further, partial resection of the inferior pedicle is essential to decompress the lateral recess successfully. The following soft tissue decompression will be difficult if the bony window is too narrow. Finally, the surgeon must confirm the lateral epidural space and free mobilization of the TNR to finish the procedure. Exposure of the TNR alone is insufficient for full-scale decompression. We can observe the TNR during the process, even early. However, surgeons must continue to decompress the exposed TNR until the neural tissue is released. Once released, the TNR begins to pulsate strongly by the arterial beat and epidural pressure.

Besides the technical considerations, knowledge of the fundamental properties of the spinal working channel endoscope is also mandatory. The visual angle is not straight but usually about 20 to 30 degrees upward. Therefore, the surgeon can obtain a wide range of visual fields by rotating the scope. There is a triangular marker at the base (6 o’clock position) so that the surgeon can recognize the bottom of the endoscopic view during the procedure. The surgical instruments usually come from the 12 o’clock direction, going to the 6 o’clock direction, and touch the central or basal position of the endoscopic visual field. Thus, the surgeon may realize the lesion is visible but difficult to reach. This discrepancy is the irony of working channel endoscopic procedures. Therefore, to manage the pathologies effectively under endoscopic visualization, the surgeon should know how to move the endoscope to place the surgical devices precisely. Steerable or articulating instruments may help treat the lesion at the corner or remote side.

### 4.4. Limitations of the Study

This study had some limitations. First, the data evaluation was performed retrospectively without an adequate control group. Therefore, selection bias may have been involved in patient inclusion. Secondly, the number of patients was too small to draw reliable conclusions. Finally, the generalizability of this study was limited by the fact that the procedures were performed by a single surgeon at a single institute. Therefore, a long-term prospective cohort study or randomized controlled trial with a larger number of patients is required to prove the effectiveness of TELLRD. However, this study suggests that percutaneous endoscopic procedures may be feasible in elderly patients with various lumbar stenoses, avoiding general anesthesia and extensive open surgery.

### 4.5. Future Perspective

As more people live longer, the number of medically compromised or old patients with symptomatic spinal stenosis will increase. Comprehensive open surgery under general anesthesia may cause significant perioperative morbidity or mortality for those patients. Therefore, the need for percutaneous endoscopic surgical techniques performed under local anesthesia is increasing. Our patient data indicated that using TELLRD for patients at risk for general anesthesia resulted in satisfactory clinical outcomes.

Theoretically, transforaminal endoscopic spine surgery may be an ideal and effective minimally invasive method while preserving normal tissues under local anesthesia. However, most standard spine surgeons are unfamiliar with this fascinating technique. They usually have limited opportunity to learn endoscopic procedures during their training period. The anatomical orientation and use of surgical instruments are quite different from open microscopic surgery. For those reasons, endoscopy technologies should be advanced to be more practical for spine surgeons to apply against actual foraminal stenosis cases. Surgical approaches, devices, and optics have evolved remarkably. The most critical point is surgical instruments specific to endoscopic surgery. Considering the inherent characteristics of rigid working-channel endoscopes, steerable or articulating devices can manage the remote or corner side pathologies. Furthermore, a systematic training program for residents and mid-career training courses should be developed. Eventually, endoscopic spine surgery techniques will be the mainstream among the spine surgeons society, according to the people’s needs.

## 5. Conclusions

Octogenarian patients with unilateral radiculopathy due to lumbar lateral recess stenosis may have a higher risk of perioperative morbidities in conventional open surgery. Full-scale lateral spinal canal decompression via a transforaminal endoscopic approach under local anesthesia is feasible and relevant for elderly or medically compromised patients. Specialized, step-by-step TELLRD techniques are essential for clinical success.

## Figures and Tables

**Table 1:** Comparison between octogenarian and younger patients.

	Octogenarian (*n* = 21)	Younger (*n* = 95)	*p* Values
Age (mean, y)	82.86 (81–88)	66.28 (42–79)	<0.0001
Male:Female	9:12	46:49	NS
Operative level			NS
L2–3	0	2	
L3–4	6	23	
L4–5	10	48	
L5–S1	5	22	
MacNab criteria			NS
Excellent	5 (23.81%)	24 (254.12%)	
Good	13 (61.90%)	60 (63.16%)	
Fair	2 (9.52%)	7 (7.37%)	
Poor	1 (4.76%)	4 (4.21%)	
Reoperation (%)	1 (open laminotomy)	3 (open laminotomy)	NS
Complication (%)			NS
Infection	0	0	
hematoma	0	1 (minor, epidural)	
dural tear	1 (minor, intraoperative)	1 (required revision)	
dysesthesia	2 (1 transient)	5 (2 transient)	

NS = not significant.

## Data Availability

The raw data supporting the conclusions of this article will be made available by the authors, without undue reservation. Data can be accessed from the corresponding author upon reasonable request.
